# Clinical spectrum of rectal cancer identifies hallmarks of early‐onset patients and next‐generation treatment strategies

**DOI:** 10.1002/cam4.5120

**Published:** 2022-08-05

**Authors:** Dingcheng Shen, Puning Wang, Yumo Xie, Zhuokai Zhuang, Mingxuan Zhu, Xiaolin Wang, Meijin Huang, Yanxin Luo, Huichuan Yu

**Affiliations:** ^1^ Guangdong Institute of Gastroenterology, Guangdong Provincial Key Laboratory of Colorectal and Pelvic Floor Disease, The Sixth Affiliated Hospital Sun Yat‐sen University Guangzhou Guangdong China; ^2^ Department of Colorectal Surgery, The Sixth Affiliated Hospital Sun Yat‐sen University Guangzhou Guangdong China

**Keywords:** cancer treatment, clinicopathological features, early‐onset, rectal cancer, young

## Abstract

**Background:**

The incidence of colorectal cancer is increasing among young adults and more rectal cancers are reported. This study aimed to identify the clinical features specific for early‐onset rectal cancer and provide insights on cancer management.

**Methods:**

Early‐onset (<50 years) and late‐onset (≥50 years) rectal cancer patients from a referral tertiary care center (SYSU cohort) and Surveillance Epidemiology and End Results database (SEER cohort) were included to perform a comprehensive comparison on clinical information.

**Results:**

A total of 552 and 80,341 patients with stages I–III rectal cancer were included in the SYSU and SEER cohorts, respectively. In the SYSU cohort, early‐onset diseases had significantly higher prevalence of family history of cancer and history of HBV infection and lower incidence of comorbidities (*p* < 0.05). In addition, early‐onset patients presented more frequently with advanced node stage (N2 stage: 16.9 vs. 9.3%, *p* = 0.017) and high‐risk features, including mucinous or signet cell carcinomas (21.8 vs. 12.9%, *p* = 0.014), poorly differentiated tumors (28.8 vs. 15.4%, *p* = 0.002), and perineural invasion (14.5 vs. 7.9%, *p* = 0.027) compared with late‐onset patients. However, early‐onset patients received more neoadjuvant (18.5 vs. 11.2%, *p* = 0.032) and adjuvant treatments (71.0 vs. 45.8%, *p* < 0.001), and they had better overall survival in both SYSU (HR 0.57, 95% CI: 0.34–0.95; *p* = 0.029) and SEER (HR 0.38, 95% CI: 0.37–0.40; *p* < 0.001) cohorts.

**Conclusion:**

Early‐onset rectal cancers are distinct from late‐onset cases in clinicopathological features, treatment modalities, and outcomes. The clinical trials and studies that are specific for young populations are needed to develop optimal strategies for cancer screening, treatment, and surveillance.

## Graphical abstract

This study including two cohorts with a total of 552 and 80,341 stages I–III rectal cancer patients demonstrated that there existed a wide set of disparities in disease history, clinicopathological features, treatment modalities, and outcomes between early‐onset and late‐onset rectal cancer patients.

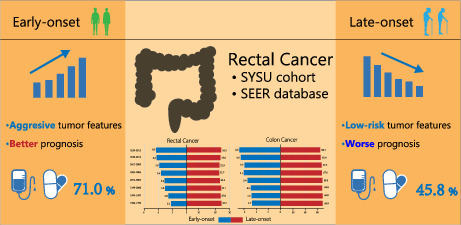

## 1 INTRODUCTION

According to the updated report on global cancer statistics, colorectal cancer is the third most common cancer and also the third causes of cancer‐related mortality worldwide.^1^, ^2^, ^3^ Although the overall morbidity and mortality of colorectal cancer have been decreasing contributed by early detection and effective treatment and surveillance, there has been an alarming increase of incidence among the populations younger than 50 years.^4^, ^5^ A large registry‐based study among 20 European countries has reported that the incidences of colorectal cancer increased with 7.9%, 4.9%, and 1.6% per year from 2004 to 2016 among individuals aged 20–29 years, 30–39 years, and 40–49 years, respectively.^6^ In addition, the proportion of rectal cancer is increasing among colorectal cancer patients in past decades,^7^ though this trend may vary in different countries such as Italy.^8^ Now, approximately 10% of newly diagnosed rectal cancer cases occur in younger adults,^9^ and it is estimated that by 2030, nearly 23% of rectal cancer will be diagnosed in individuals aged <50 years.^10^

Although early‐onset disease is in part related to hereditary cancer syndromes, the majority are sporadic with uncertain risk factors.^11^ Previous studies reported that early‐onset colorectal cancer patients are clinically, pathologically, and molecularly distinct from late‐onset cases.^12^, ^13^, ^14^, ^15^, ^16^, ^17^ However, the characteristics remain to be specifically described for rectal cancer in early‐onset group to provide treatment and surveillance strategies for this distinct group of patients with increasing incidence.

We, therefore, focused on early‐onset rectal cancer and performed a comprehensive characterization of its clinicopathological features by comparing it with late‐onset disease in the Surveillance, Epidemiology, and End Results (SEER) database and our in‐house cohort with wide populations to provide an insight on the next‐generation screen, treatment, and surveillance for the next‐generation patients.

## 2 METHODS

### 2.1 Patient cohorts

A consecutive series of 18,013 colorectal cancer patients in a large referral tertiary care center were identified and screened between June 2007 and June 2012. The patient data, including demographic and clinicopathological characteristics and follow‐up data, were collected from the prospectively maintained institutional database program of colorectal disease (IDPCD) as introduced previously.^18^, ^19^ The patients with hereditary colorectal diseases, including familial adenomatous polyposis (FAP) and hereditary nonpolyposis tumors, were excluded. As a result, a total of 552 pathologically confirmed stages I to III rectal cancers undergoing curative‐intent resection were finally included in the Sun Yat‐sen University (SYSU) cohort. Patients were staged according to the seventh edition of the AJCC clinical staging system.^20^ Moreover, we obtained the clinicopathological characteristics of rectal cancer patients with stages I to III stage between 1992 and 2015 from the SEER database (Incidence SEER Research Date, 9 Registries, November 2020 Sub [from 1975–2018])^21^ to validate the findings in the SYSU cohort and investigate the trend in the morbidity of colon and rectal cancers in various age groups. Patients were divided into early‐onset and late‐onset groups according to the age cutoff of 50 years, which was well characterized and defined in the previous studies.^6^, ^7^, ^8^, ^12^, ^13^, ^14^, ^15^, ^16^, ^17^ The patient disposition flow is described in Figure S1.

### 2.2 Treatment, follow‐up, and study endpoints

According to the NCCN Guideline for Rectal Cancer, patients in SYSU cohort were treated and followed up with the details described in previous publications.^22^, ^23^, ^24^, ^25^ Briefly, after surgery, patients were observed every 3–6 months for the first 2 years and every 6 months for the next 3 years. Routine visits include pertinent medical history, medical examination, and measurement of serum CEA and CA19‐9 levels. Radiologic examinations consisting of chest and abdominopelvic CT were scheduled every 6–12 months after surgery for a total of 5 years, and colonoscopy was scheduled 1 year after surgery and repeated in 1–3 years. The primary endpoints were disease‐free survival (DFS) and overall survival (OS). DFS was calculated from the date of curative resection to the date of recurrence, death, or censored date. OS was defined as the time from the date of curative resection to death or censoring.

### 2.3 Statistical analysis

Continuous variables were described as median and interquartile range and were compared using the Mann–Whitney *U* test because of their abnormal distribution. Categorical variables were reported as numbers with percentages, and they were assessed using the chi‐square test or the Fisher exact test where appropriate. DFS and OS were calculated using the Kaplan–Meier method and performed through log‐rank test between group comparisons. Univariate survival analysis for OS and DFS was evaluated using a Cox proportional hazards model. The statistical analyses were performed through SPSS 22.0 and R‐project 3.5.1. A two‐sided *p* value <0.05 was regarded with statistical significance.

## 3 RESULTS

### 3.1 Overview of patient cohorts

**FIGURE 1:**
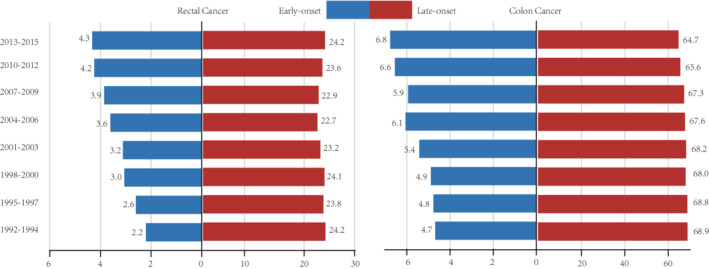
Time trends of proportion of early‐ and late‐onset colon or rectal cancer in the pooled Surveillance Epidemiology and End Results database (SEER). The bar charts showed the proportions of younger rectal and colon cancer cases were increasing year by year, while they decreasing in late‐onset colon cancer patients.

In the SYSU cohort, a total of 552 rectal cancer patients with stages I–III were included. There were 322 male (58.3%) and 230 female (41.7%) patients, with a median age of 59 years (21–89 years). The TNM staging among those patients was distributed as 25.5%, 32.8%, and 41.7% for stages I, II, and III, respectively. Among them, there were 124 (22.5%) early‐onset and 428 (77.5%) late‐onset rectal cancer cases, with a median age at the time of diagnosis at 39 years (21–49 years) and 64 years (50–89 years), respectively. With a median follow‐up time of 46.0 (35.0–61.8) months, the DFS rates at 1, 3, and 5 years were 87.1%, 71.2%, and 66.0% and the OS rates at 1, 3, and 5 years were 96.7%, 84.1.0%, and 76.1%. In the SEER cohort, the early‐onset cases were substantially less than late‐onset cases, though the proportion of early‐onset cases was increasing per year in the past decades (Figure 1). Moreover, a total of 80,341 cases with stages I–III rectal cancer were included in the SEER cohort, including 11.8% of early‐onset patients and 88.2% of late‐onset patients.

### 3.2 Early‐onset patients are distinct from late‐onset patients in family history and comorbidity

**FIGURE 2:**
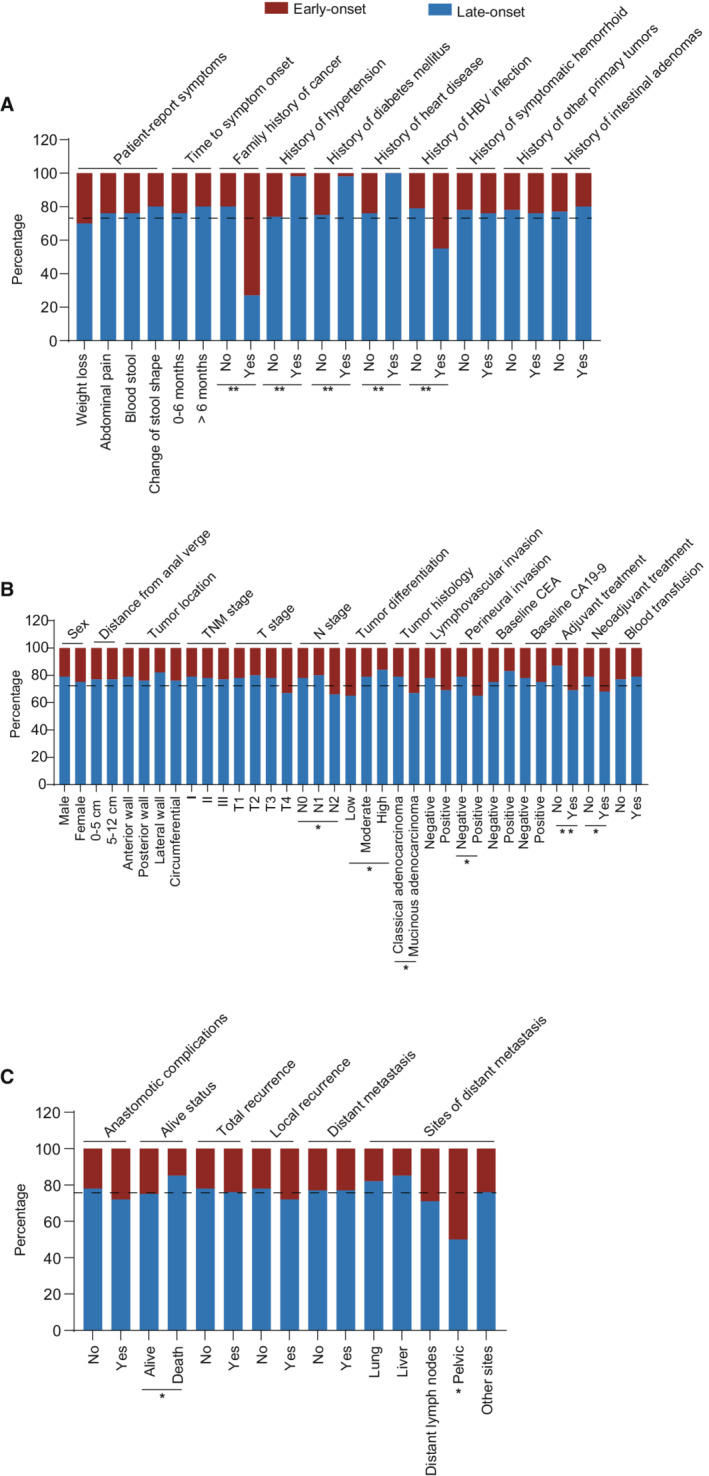
Comprehensive comparison on clinical information between early‐onset and late‐onset rectal cancer patients. (A) Characteristics of history between early‐onset and late‐onset rectal cancer patients. (B) Distribution of baseline characteristics between early‐onset and late‐onset rectal cancer patients. (C) Short‐term and long‐term outcomes after curative resection in early‐onset and late‐onset rectal cancers. *p value was <0.05 and considered statistically significant.

The disease history of early‐ and late‐onset patients in the SYSU cohort is summarized in Figure 2A and Table S1. The most common presenting symptoms were blood stool (80.6 vs. 72.9%), change of stool shape (50.8 vs. 59.8%), abdominal pain (12.9 vs. 11.7%), and weight loss (11.3 vs. 7.5%) in both early‐onset and late‐onset patients. There were no statistical differences in patient‐reported symptoms and time to treatment from symptom onset between the two groups. Consistent with previous findings,^26^, ^27^ early‐onset patients were significantly associated with a family history of cancer (8.9 vs. 0.9%; *p* < 0.001.) Interestingly, more patients with history of hepatitis B virus (HBV) infection were found in the early‐onset group (10.5 vs. 3.7%; *p* = 0.003). As expected, late‐onset cases were more susceptible to age‐related comorbidities, including hypertension (1.6 vs. 20.3%), diabetes mellitus (0.8 vs. 12.1%), and heart diseases (0 vs. 7.0%) (all *p* < 0.05).

### 3.3 Early‐onset diseases are characterized by high‐risk clinicopathological features with more cancer treatment

Early‐onset patients tended to have negative baseline CEA levels compared to late‐onset patients, though the difference is not significant (79.2 vs. 70.1%, *p* = 0.051). There was no significant difference in circumferential tumor location between early‐onset and late‐onset rectal cancer patients in the SYSU cohort. The early‐onset patients had higher incidence of low tumor differentiation (28.8 vs. 15.4%; *p* = 0.002), mucinous and signet ring rectal carcinoma (21.8 vs. 12.9%; *p* = 0.014), and presented more with advanced N stage (N2 stage: 16.9 vs. 9.3%; *p* = 0.017) in SYSU cohort (Figure 2B and Table S2). These findings were further validated in the SEER cohort (all *p* < 0.05, Table S3). Together, early‐onset patients had more advanced and aggressive tumors in both cohorts. When it comes to treatment, early‐onset patients got more access to neoadjuvant (18.5 vs. 11.2%; *p* = 0.032) and adjuvant (71.0 vs. 45.8%; *p* < 0.001) chemo(radio)therapy (Table S2).

### 3.4 Short‐ and long‐term treatment outcomes of early‐onset patients

The overall incidence of postoperative anastomotic complications, including any disease of anastomotic leakage, bleeding, and stenosis, did not differ significantly between two age groups (*p* = 0.280) in the SYSU cohort.

**FIGURE 3:**
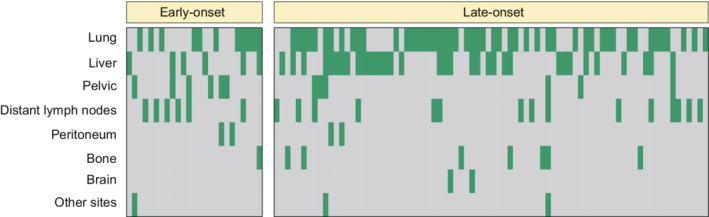
Matrix plot of metastatic sites after curative‐intent treatment in early‐onset and late‐onset rectal cancers. The matrix plot showed the distribution of different distant metastasis organs among metachronous metastatic disease in early‐onset and late‐onset patients. Each column was labeled with a patient (green column, patient with metastasis; gray column, patients without metastasis).

**FIGURE 4:**
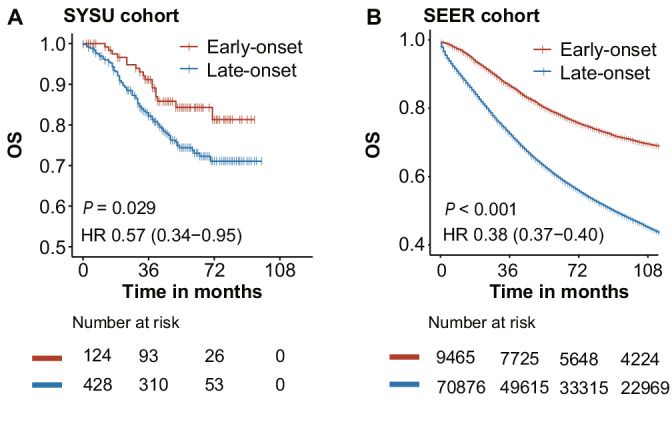
Survival outcomes of early‐onset and late‐onset rectal cancers in two cohorts. The Kaplan–Meier curves showed that early‐onset rectal cancer patients seem to have better overall survival (OS) in our cohort (A) and the SEER cohort (B) when compared with late‐onset patients. The log‐rank test was applied and *p* value was given in each plot.

The local recurrence and distant metastasis after curative‐intent treatment were detected in 12.3% and 19.7% of early‐onset patients (*p* = 0.350) and were detected in 9.4% and 19.5% of the late‐onset patients (*p* = 0.970) in the SYSU cohort (Figure 2C and Table S4). Among the patients with metachronous metastasis, the site matrix plot represented that early‐onset patients were characterized by a higher incidence of pelvic (4.8% vs. 1.4%, *p* = 0.021) metastasis compared with late‐onset cases (Figure 3). The Kaplan–Meier analysis revealed that early‐onset rectal cancer patients had better OS when compared with late‐onset patients in both SYSU (hazard ratio 0.57, 95% CI: 0.34–0.95; *p* = 0.029) and SEER (hazard ratio 0.38, 95% CI: 0.37–0.40; *p* < 0.001) cohorts (Figure 4).

**FIGURE 5:**
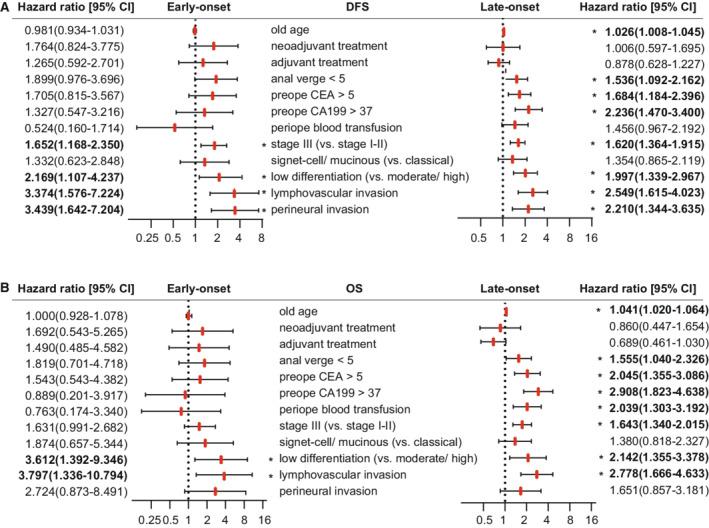
Forrest plots of prognostic factors in early‐onset and late‐onset rectal cancers. Forrest plots for univariate analysis of prognostic factors for disease‐free survival DFS (A) and overall survival OS (B) were shown in the early‐onset (left) and late‐onset (right) rectal cancer patients. *p value was <0.05 and considered statistically significant.

The univariate Cox analysis for survival showed the prognostic factors of two age groups in the SYSU cohort (Figure 5). Advanced stage, low tumor differentiation, lymphovascular invasion, and perineural invasion were significant risk factors for DFS or OS in both early‐onset and late‐onset rectal cancer patients, while the other significant risk factors for late‐onset patients, including older age, distance from the anal verge to tumor <5 cm, and elevated CEA or CA19‐9, were not significant for early‐onset patients.

## 4 DISCUSSION

In this study, we demonstrated that there existed a wide set of disparities in disease history, clinicopathological features, treatment modalities, and clinical outcomes between early‐onset and late‐onset non‐metastatic rectal cancer patients. In brief, early‐onset patients had more family history of cancer and history of HBV infection, and they were more likely to exhibit regional lymph nodes metastasis, poor differentiation, mucinous or signet ring carcinoma, and perineural invasion at initial diagnosis and present with pelvic metastasis after treatment. We also found that early‐onset rectal cancer patients received more neoadjuvant and adjuvant treatments, and they had favorable survival outcomes, which were externally validated in the SEER cohort. In addition, we demonstrated that advanced stage, low tumor differentiation, lymphovascular invasion, and perineural invasion were significantly associated with survival outcomes in early‐onset patients. The keynote of the hallmarks of early‐onset rectal cancer is briefly summarized in Table 1. Considering these distinct clinical features and outcomes, it is necessary to consider age as a crucial factor prior to design studies for rectal cancer.

**TABLE 1:** Keynote of the hallmarks of early‐onset rectal cancer

Clinical characteristics	Early onset	Late onset	References
Family history of cancer	**√**		[^26^, ^27^, ^42^]
HBV infection	**√**		[^30^]
Advanced stage	**√**		[^13^, ^42^, ^43^]
Lymph nodes metastasis	**√**		[^13^, ^15^]
Low differentiation	**√**		[^12^, ^13^, ^42^]
Mucinous/signet cell carcinoma	**√**		[^12^, ^13^, ^42^]
Elevated baseline CEA		**√**	Not found
Aggressive chemo(radio)therapy	**√**		[^13^, ^17^, ^40^, ^42^, ^43^]
Better survival outcome	**√**		[^13^, ^17^, ^42^]

The previous epidemiological studies have shown that the early‐onset colorectal cancer is increasing worldwide.^9^, ^28^ In the current SEER cohort analysis, we found that the early‐onset rectal cancer was the group with incidence increasing most obviously in the past decades. This alarming trend highlights the clinical significance of characterizing early‐onset rectal cancer patients in the current study.

A better understanding of the underlying mechanism for the disparities between early‐onset and late‐onset cancers will facilitate the development of specific prevention and treatment strategies for this population. Interestingly, the early‐onset cases were more likely to have a history of HBV infection in our cohort, which is consistent with the previous age‐specific analysis that the HBV‐related colorectal cancer risk decreases with aging in the HBV endemic regions.^29^, ^30^ The mechanism underlying the link between HBV infection and colorectal carcinogenesis has not been well understood at this moment. Previous studies have indicated that HBV may exist in extrahepatic tissues, such as the kidneys, lymph nodes, pancreas, and gastrointestinal tract.^31^, ^32^, ^33^ Therefore, we speculate that the role of HBV in the oncogenesis of colorectal cancer may be explained with the well‐documented mechanisms of HBV‐induced hepatocellular cancer, such as integration of HBV DNA into the host genome, alteration of the expression and signaling pathway of the host gene,^34^ and the persistent inflammation, hypoxia, angiogenesis, and oxidative stress brought from the chronic infection of HBV.^35^, ^36^ We, therefore, proposed that the underlying molecular basis for the oncogenic role of HBV infection in the early‐onset rectal cancer is of significance to be further investigated.

In the current study, we found that the early‐onset group had more advanced node‐stage tumors compared with the late‐onset group. This may be a result of the achievement of early detection contributed by the cancer screen protocol with colonoscopy and FIT tests that have been widely applied in the populations older than 50. Our findings support the recommendation from the American Cancer Society that the initiation age of cancer screening was extended from 50 to 45 years.^37^

Although the consensus of specific treatment for early‐onset colorectal cancer has not been well developed, patients with high‐risk feature are recommended for more chemo(radio)therapy according to the treatment protocols from NCCN guideline.^24^ In addition, the reductions in organ function and unfavorable preexisting comorbidities in the old populations make late‐onset patients less tolerated with chemo(radio)therapy,^38^ which may also lead to this treatment difference between age groups. Furthermore, this disparity can also be partially explained by concern surrounding the safety and efficacy of systemic treatment regarding the paucity of data from high evidence‐based clinical studies in older adult populations.^39^ Our findings were consistent with a nationwide comparative cohort study from the American College of Surgeons Commission on Cancer showing that early‐onset cases were two to four times more likely to receive postoperative systemic chemotherapy than older cases in each disease‐stage group with colon cancer.^40^

It is widely acknowledged that standard neoadjuvant and adjuvant therapies administered to colorectal cancer patients could significantly improve their oncological outcomes.^41^ It sounds rational that early‐onset patients with more advanced tumors would receive more chemo(radio)therapy, and thus, they would get survival benefit from the treatment. In the current study, we found that early‐onset cases had better survival outcome than late‐onset cases in the both two cohorts. It seems that the chemo(radio)therapy not only neutralized the unfavorable effect from the advanced stage and high‐risk tumor features, but also brought additional survival benefit for early‐onset cases. The survival benefit from chemo(radio)therapy was also found in the US,^13^ Netherlandish^42^ and Irish^17^ cohorts with early‐onset colorectal cancer. Our findings together with these studies support the active use of systemic neoadjuvant or adjuvant treatments for early‐onset individuals. However, a retrospective analysis of a cohort from the National Cancer Data Base of the US found that the NCCN guideline‐driven multimodal regimen for stages II and III disease was not associated with a survival benefit for early‐onset cases.^43^ With regard to these inconsistent findings, we propose that the clinical trials in early‐onset cases are needed to develop cancer treatment strategies that are specific for young populations.

Although the two cohorts with sufficient cases could provide robust evidence to support our findings, this study has some limitations. First, this study was limited by its retrospective nature, and the specific results derived from the single‐center analysis may not be generalized to other populations. Second, the sample size of early‐onset cases in the SYSU cohort was relatively small, which makes it difficult to further split the early‐onset cases into younger groups, such as patients aged <40 and 30 years, to conduct a robust statistical analysis. Then, the details about neoadjuvant regimens and response to neoadjuvant treatment were not well documented in SYSU cohort, which limits the in‐depth analysis in this study. Finally, we focused on rectal cancer in this study, while future studies are of interest to specifically compare the early‐onset and late‐onset colon cancers to provide valuable sources for next‐generation management of colon cancer. As a result, the optimal multimodal regimen and surveillance strategies specific for early‐onset colorectal cancers should be confirmed in the future clinical trials.

## 5 CONCLUSION

This study characterized the demographic and clinicopathological features of early‐onset rectal cancer, an increasingly diagnosed subgroup of colorectal cancer patients, by comparing them with late‐onset rectal cancer patients. We found that early‐onset patients were more likely to have advanced node staged tumors and high‐risk pathological features, including low differentiation, mucinous or signet cell carcinoma, and perineural invasion. However, early‐onset patients got more access to neoadjuvant and adjuvant treatment with better survival outcomes compared with late‐onset patients. Our findings indicated that this survival advantage might be attributed to the more sufficient treatment early‐onset patients received. Considering that early‐onset rectal cancer had distinct clinical and pathological features, the specific clinical trials and studies for young populations are needed to develop optimal strategies for cancer screening, treatment, and surveillance.

## AUTHOR CONTRIBUTIONS

Study concept and design: DS, YL, and HY. Acquisition of data: MZ, PW, XW, and MH. Analysis and interpretation of data: DS, YX, ZZ, and PW. Drafting of the manuscript: DS and HY. Critical revision of the manuscript for important intellectual content: all authors. All authors had access to the study data and reviewed and approved the final manuscript.

## CONFLICT OF INTEREST

The authors declare that they have no conflicts of interest.

## ETHICS STATEMENT

The study protocol was reviewed and approved by the Institutional Review Board of the Sixth Affiliated Hospital of Sun Yat‐sen University (SYSU) (no. 2017ZSLYEC‐006), and written informed consent was obtained from all subjects or their representatives for the study participation. The study was performed in accordance with the Declaration of Helsinki.

## Supporting information


Figure S1

Table S1

## Data Availability

The data from the SYSU cohort that support the findings of this study are available on request from the corresponding author. The data are not publicly available due to privacy or ethical restrictions. The data that support the findings of this study are openly available in the Surveillance, Epidemiology, and End Results database.
