# Supplementary figures and images for: Clinical spectrum of rectal cancer identifies hallmarks of early‐onset patients and next‐generation treatment strategies
Source: Cancer Med. 2022 Aug 5;12(3):3433–41. doi: 10.1002/cam4.5120 (PMC9939204; doi:10.1002/cam4.5120)

**Figure S1**

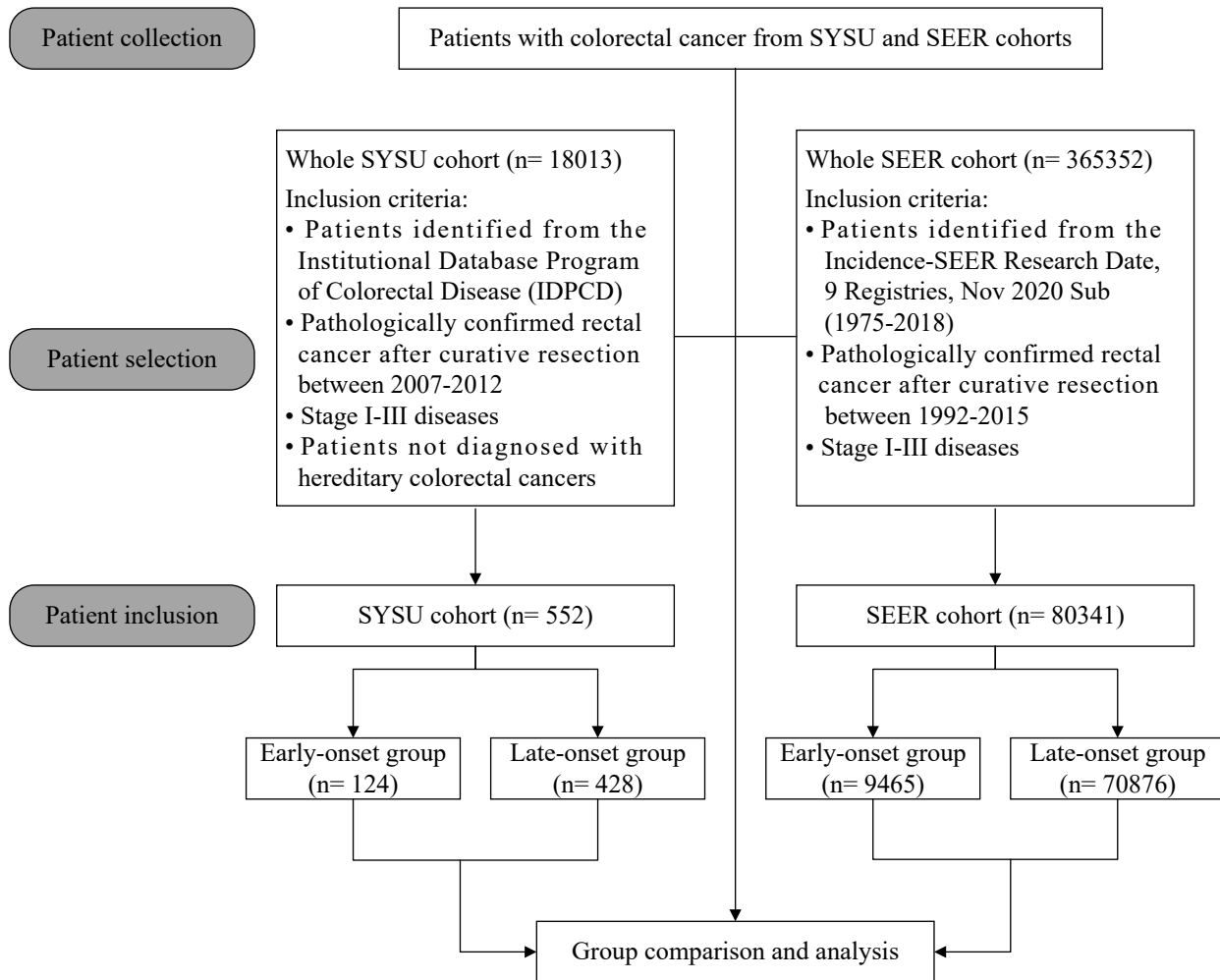

Supplement: Supplementary file 1 — Figure S1 [file CAM4-12-3433-s001.pdf]
