# Supplementary material for: Clinical spectrum of rectal cancer identifies hallmarks of early‐onset patients and next‐generation treatment strategies
Source: Cancer Med. 2022 Aug 5;12(3):3433–41. doi: 10.1002/cam4.5120 (PMC9939204; doi:10.1002/cam4.5120)
Supplement: Supplementary file 2 — Table S1 [file CAM4-12-3433-s002.docx]

**Table S1 Characteristics of history between early-onset and late-onset rectal cancer patients**

| Characteristic | Overall population | Onset | | *P* value |
| --- | --- | --- | --- | --- |
|  |  | Early-onset | Late-onset |  |
|  | (N=552) | (N=124) | (N=428) |  |
| **Family history of cancer** |  |  |  | **< 0.001** |
| Yes | 15 (2.7) | 11 (8.9) | 4 (0.9) |  |
| No | 537 (97.3) | 113 (91.1) | 424 (99.1) |  |
| **Patient-reported symptoms*** |  |  |  |  |
| Weight loss | 46 (8.3) | 14 (11.3) | 32 (7.5) | 0.176 |
| Abdominal pain | 66 (12.0) | 16 (12.9) | 50 (11.7) | 0.712 |
| Blood stool | 412 (74.6) | 100 (80.6) | 312 (72.9) | 0.081 |
| Change of stool shape | 319 (57.8) | 63 (50.8) | 256 (59.8) | 0.074 |
| **Time to symptom onset** |  |  |  |  |
| Continuous variable (months) | 4.0 (2.0-12.0) | 4.0 (2.0-7.5) | 5.0 (2.0-12.0) | 0.214 |
| 0-6 months | 370 (68.6) | 88 (72.7) | 282 (67.5) | 0.272 |
| > 6 months | 169 (31.4) | 33 (27.3) | 136 (32.5) |  |
| **History of hypertension** | |  |  | **< 0.001** |
| No | 463 (83.9) | 122 (98.4) | 341 (79.7) |  |
| Yes | 89 (16.1) | 2 (1.6) | 87 (20.3) |  |
| **History of diabetes mellitus** |  |  |  | **< 0.001** |
| No | 499 (90.4) | 123 (99.2) | 376 (87.9) |  |
| Yes | 53 (9.6) | 1 (0.8) | 52 (12.1) |  |
| **History of heart disease** |  |  |  | **0.002** |
| No | 522 (94.6) | 124 (100) | 398 (93.0) |  |
| Yes | 30 (5.4) | 0 (0) | 30 (7.0) |  |
| **History of HBV infection** |  |  |  | **0.003** |
| No | 523 (94.7) | 111 (89.5) | 412 (96.3) |  |
| Yes | 29 (5.3) | 13 (10.5) | 16 (3.7) |  |
| **History of symptomatic hemorrhoid** |  |  |  | 0.740 |
| No | 507 (91.8) | 113 (91.1) | 394 (92.1) |  |
| Yes | 45 (8.2) | 11 (8.9) | 34 (7.9) |  |
| **History of other primary tumors** |  |  |  | 0.747 |
| No | 494 (89.5) | 110 (88.7) | 384 (89.7) |  |
| Yes | 58 (10.5) | 14 (11.3) | 44 (10.3) |  |
| **History of intestinal adenomas** |  |  |  | 0.540 |
| No | 476 (86.2) | 109 (87.9) | 367 (85.7) |  |
| Yes | 76 (13.8) | 15 (12.1) | 61 (14.3) |  |

* Not mutually exclusive

**Table S2 Distribution of baseline characteristics between early-onset and late-onset rectal cancer patients**

| Characteristic | Overall population | Onset | | *P* value |
| --- | --- | --- | --- | --- |
|  |  | Early-onset | Late-onset |  |
|  | (N=552) | (N=124) | (N=428) |  |
| **Age-yr, median(range)** | 59 (21-89) | 39 (21-49) | 64 (50-89) | **<0.001** |
| **BMI, median(range)** | 22.1 (8.3-35.9) | 22.0 (8.3-30.2) | 22.2 (13.3-35.9) | 0.655 |
| **Sex** |  |  |  | 0.270 |
| Male | 322 (58.3) | 67 (54.0) | 255 (59.6) |  |
| Female | 230 (41.7) | 57 (46.0) | 173 (40.4) |  |
| **Tumor location: Distance from anal verge** | |  |  | 0.995 |
| 0-5 cm | 207 (38.5) | 47 (38.5) | 160 (38.6) |  |
| 5-12 cm | 330 (61.5) | 75 (61.5) | 255 (61.4) |  |
| **Tumor location: Orientation** | |  |  | 0.902 |
| Anterior wall | 72 (13.0) | 15 (12.1) | 57 (13.3) | 0.722 |
| Posterior wall | 94 (17.0) | 23 (18.5) | 71 (16.6) | 0.609 |
| Lateral wall | 50 (9.1) | 9 (7.3) | 41 (9.6) | 0.428 |
| Circumferential | 139 (25.2) | 34 (27.4) | 105 (24.5) | 0.514 |
| **TNM Stage, AJCC** |  |  |  | 0.876 |
| I | 141 (25.5) | 30 (24.2) | 111 (25.9) | 0.695 |
| II | 181 (32.8) | 40 (32.3) | 141 (32.9) | 0.884 |
| III | 230 (41.7) | 54 (43.5) | 176 (41.1) | 0.629 |
| **T stage** |  |  |  | 0.389 |
| T1 | 54 (9.8) | 12 (9.7) | 42 (9.8) | 0.964 |
| T2 | 113 (20.5) | 23 (18.5) | 90 (21.0) | 0.546 |
| T3 | 343 (62.1) | 75 (60.5) | 268 (62.6) | 0.666 |
| T4 | 42 (7.6) | 14 (11.3) | 28 (6.5) | 0.079 |
| **N stage** |  |  |  | **0.051** |
| N0 | 322 (58.3) | 70 (56.5) | 252 (58.9) | 0.629 |
| N1 | 169 (30.6) | 33 (26.6) | 136 (31.8) | 0.272 |
| N2 | 61 (11.1) | 21 (16.9) | 40 (9.3) | **0.017** |
| **Tumor differentiation** |  |  |  | **0.002** |
| Low | 98 (18.4) | 34 (28.8) | 64 (15.4) | 0.001 |
| Moderate | 296 (55.4) | 61 (51.7) | 235 (56.5) | 0.261 |
| High | 140 (26.2) | 23 (19.5) | 117 (28.1) | 0.048 |
| **Tumor histology** |  |  |  | **0.014** |
| Classical adenocarcinoma | 470 (85.1) | 97 (78.2) | 373 (87.1) |  |
| Signet-ring cell carcinoma / Mucinous adenocarcinoma | 82 (14.9) | 27 (21.8) | 55 (12.9) |  |
| **Lymphovascular invasion** |  |  |  | 0.152 |
| Negative | 503 (91.1) | 109 (87.9) | 394 (92.1) |  |
| Positive | 49 (8.9) | 15 (12.1) | 34 (7.9) |  |
| **Perineural invasion** |  |  |  | **0.027** |
| Negative | 500 (90.6) | 106 (85.5) | 394 (92.1) |  |
| Positive | 52 (9.4) | 18 (14.5) | 34 (7.9) |  |
| **Preoperative CEA** |  |  |  | **0.051** |
| 0-5 ng/mL | 381 (72.2) | 95 (79.2) | 286 (70.1) |  |
| > 5 ng/mL | 147 (27.8) | 25 (20.8) | 122 (29.9) |  |
| **Preoperative CA19-9** |  |  |  | 0.574 |
| 0-37 ng/mL | 452 (86.4) | 101 (84.9) | 351 (86.9) |  |
| > 37 ng/mL | 71 (13.6) | 18 (15.1) | 53 (13.1) |  |
| **Adjuvant treatment** |  |  |  | **<0.001** |
| No | 268 (48.6) | 36 (29.0) | 232 (54.2) |  |
| Yes | 284 (51.4) | 88 (71.0) | 196 (45.8) |  |
| **Neoadjuvant treatment** |  |  |  | **0.032** |
| No | 481 (87.1) | 101 (81.5) | 380 (88.8) |  |
| Yes | 71 (12.9) | 23 (18.5) | 48 (11.2) |  |
| **Blood Transfusion** |  |  |  | 0.783 |
| No | 463 (83.9) | 105 (84.7) | 358 (83.6) |  |
| Yes | 89 (16.1) | 19 (15.3) | 70 (16.4) |  |

**Table S3 Distribution of baseline characteristics between early-onset and late-onset rectal cancer patients in the SEER cohort.**

| Characteristic | Overall population | | | Onset | | *P* value |
| --- | --- | --- | --- | --- | --- | --- |
|  |  |  |  | Early-onset | Late-onset |  |
|  | (N=80341) | | | (N=9465) | (N=70876) |  |
| **Age-yr, median(range)** | 63 (20-86) | | | 44 (20-49) | 69 (50-85) | **< 0.001** |
| **Sex** |  | | |  |  | **0.002** |
| Male | 45865 (57.1) | | | 5264 (55.6) | 40601 (57.3) |  |
| Female | 34476 (42.9) | | | 4201 (44.4) | 30275 (42.7) |  |
| **TNM Stage, AJCC** |  | | |  |  | **< 0.001** |
| I | 31065 (38.7) | | | 2900 (30.6) | 28165 (39.7) | < 0.001 |
| II | 22939 (28.6) | | | 2351 (24.8) | 20588 (29.0) | < 0.001 |
| III | 26337 (32.7) | | | 4214 (44.5) | 22123 (31.2) | < 0.001 |
| **T stage** | |  |  | |  | **< 0.001** |
| T1 | | 4418 (5.5) | 463 (4.3) | | 3955 (5.6) | 0.006 |
| T2 | | 3085 (3.8) | 396 (4.2) | | 2689 (3.8) | 0.063 |
| T3 | | 9646 (12.0) | 1576 (16.7) | | 8070 (11.4) | < 0.001 |
| T4 | | 1737 (2.2) | 285 (3.0) | | 1452 (2.0) | < 0.001 |
| Unknown | | 61455 (76.5) | 6745 (71.8) | | 54710 (77.2) |  |
| **N stage** | |  |  | |  | **< 0.001** |
| N0 | | 11480 (14.3) | 1298 (13.7) | | 10182 (14.4) | 0.089 |
| N1 | | 5669 (7.1) | 988 (10.4) | | 4681 (6.6) | < 0.001 |
| N2 | | 1841 (2.3) | 454 (4.8) | | 1378 (1.9) | < 0.001 |
| Unknown | | 61351 (76.3) | 6725 (71.1) | | 54626 (77.1) |  |
| **Tumor differentiation** |  | | |  |  | **0.002** |
| Low | 11348 (14.1) | | | 1586 (16.8) | 9762 (13.8) | < 0.001 |
| Moderate | 54689 (68.1) | | | 6336 (66.9) | 48353 (68.2) | 0.012 |
| High | 6641 (8.3) | | | 672 (7.1) | 5969 (8.4) | < 0.001 |
| Unknown | 7663 (9.5) | | | 871 (9.2) | 6792 (9.6) |  |
| **Tumor histology** |  | | |  |  | **< 0.001** |
| Classical adenocarcinoma | 73479 (91.5) | | | 8455 (89.3) | 65024 (91.7) |  |
| Signet-ring cell carcinoma / Mucinous adenocarcinoma | 5249 (6.5) | | | 770 (8.1) | 4479 (6.3) |  |
| Unknown | 1613 (2.0) | | | 240 (2.6) | 1373 (2.0) |  |

**Table S4 Short-term and long-term outcomes after curative resection in early-onset and late-onset rectal cancers**

| **Outcomes** | All population | Early onset | Late onset |  |
| --- | --- | --- | --- | --- |
|  | (N=552) | (N=124) | (N=428) | *P*-value |
| **Anastomotic complications^1^** |  |  |  | 0.280 |
| No | 483 (87.5) | 105 (84.7) | 378 (88.3) |  |
| Yes | 69 (12.5) | 19 (15.3) | 50 (11.7) |  |
| **Alive status** |  |  |  | **0.023** |
| Alive | 436 (79.0) | 107 (86.3) | 329 (76.9) |  |
| Death | 116 (21.0) | 17 (13.7) | 99 (23.1) |  |
| **Total recurrence^2^** |  |  |  | 0.686 |
| No | 404 (73.2) | 89 (71.8) | 315 (73.6) |  |
| Yes | 148 (26.8) | 35 (28.2) | 113 (26.4) |  |
| **Local recurrence** |  |  |  | 0.350 |
| No | 483 (89.9) | 107 (87.7) | 376 (90.6) |  |
| Yes | 54 (10.1) | 15 (12.3) | 39 (9.4) |  |
| **Distant metastasis** |  |  |  | 0.970 |
| No | 432 (80.4) | 98 (80.3) | 334 (80.5) |  |
| Yes | 105 (19.6) | 24 (19.7) | 81 (19.5) |  |
| **Sites of distant metastasis** |  |  |  |  |
| Lung | 60 (10.9) | 11 (8.9) | 49 (11.4) | 0.417 |
| Liver | 40 (7.2) | 6 (4.8) | 34 (7.9) | 0.240 |
| Distant lymph nodes | 21 (3.8) | 6 (4.8) | 15 (3.5) | 0.494 |
| Pelvic | 12 (2.2) | 6 (4.8) | 6 (1.4) | **0.021** |
| Other sites | 17 (3.1) | 4 (3.2) | 13 (3.0) | 0.916 |

1. Anastomotic complications in this study include any disease of anastomotic leakage, bleeding, and stenosis reported at the postoperative and follow-up phase; 2. Recurrence in this study refers to local recurrence and/or distant metastasis after curative resection.
